# In Vivo and In Vitro Assays Evaluating the Biological Activity of Taurine, Glucose and Energetic Beverages

**DOI:** 10.3390/molecules26082198

**Published:** 2021-04-11

**Authors:** Marcos Mateo-Fernández, Fernando Valenzuela-Gómez, Rafael Font, Mercedes Del Río-Celestino, Tania Merinas-Amo, Ángeles Alonso-Moraga

**Affiliations:** 1 Department of Genetics, University of Córdoba, 14071 Córdoba, Spain; tania.meram@gmail.com (T.M.-A.); ge1almoa@uco.es (Á.A.-M.); 2 Agri-Food Laboratory, Avda. Menéndez Pidal, s/n, 14080 Córdoba, Spain; rafaelm.font@juntadeandalucia.es (R.F.); mercedes.rio.celestino@juntadeandalucia.es (M.D.R.-C.)

**Keywords:** classic Red Bull^®^, taurine, glucose, *Drosophila melanogaster*, HL-60 cell line

## Abstract

Taurine is one of the main ingredients used in energy drinks which are highly consumed in adolescents for their sugary taste and stimulating effect. With energy drinks becoming a worldwide phenomenon, the biological effects of these beverages must be evaluated in order to fully comprehend the potential impact of these products on the health due to the fact nutrition is closely related to science since the population consumes food to prevent certain diseases. Therefore, the aim of this study was to evaluate the biological effects of taurine, glucose, classic Red Bull^®^ and sugar-free Red Bull^®^ in order to check the food safety and the nutraceutical potential of these compounds, characterising different endpoints: (i) Toxicology, antitoxicology, genotoxicology and life expectancy assays were performed in the *Drosophila melanogaster* model organism; (ii) The in vitro chemopreventive activity of testing compounds was determined by assessing their cytotoxicity, the proapoptotic DNA-damage capability to induce internucleosomal fragmentation, the strand breaks activity and the modulator role on the methylation status of genomic repetitive sequences of HL-60 promyelocytic cells. Whereas none tested compounds showed toxic or genotoxic effect, all tested compounds exerted antitoxic and antigenotoxic activity in *Drosophila*. Glucose, classic Red Bull^®^ and sugar-free Red Bull^®^ were cytotoxic in HL-60 cell line. Classic Red Bull^®^ induced DNA internucleosomal fragmentation although none of them exhibited DNA damage on human leukaemia cells. In conclusion, the tested compounds are safe on *Drosophila melanogaster* and classic Red Bull^®^ could overall possess nutraceutical potential in the in vivo and in vitro model used in this study. Besides, taurine could holistically be one of the bioactive compounds responsible for the biological activity of classic Red Bull^®^.

## 1. Introduction

Energy drinks are caffeinated soft drinks which adolescents consume for the taste and stimulating effect. Energy drink intake is increasing all over the world as well as the scientific studies related to their effects [1,2]. Manufacturers of these beverages encourage their consumption with statements claiming a diversity of benefits [3], stating that these products are appropriate for consumers and free of danger. However, among scientists the food safety of these beverages is controversial [4]. With energy drinks becoming a worldwide phenomenon, the biological effects of these beverages must be evaluated in order to fully comprehend the potential impact of these products on health.

Taurine (TAU) is one of the main ingredients used in energy drinks such as Classic Red Bull^®^ (CRB) and Sugar Free Red Bull^®^ (SFRB). TAU is a natural amino acid that can be synthesised from methionine and cysteine mainly within the liver, kidney, astrocytes or testis [5]. However, dietary taurine supplementation may be required to achieve optimal taurine status in newborn infants [6] playing an important role in human during development, bile acid conjugation, osmoregulation, detoxification of xenobiotics, cell membrane stabilization, modulation of cellular calcium flux, modulation of neuronal excitability, antioxidant and anti-inflammatory properties among others [7]. Therefore, TAU is an essential amino-acid end-product. TAU has been extensively studied because of the variable evidence for the beneficial effects of this amino acid food supplementation [8].

Glucose (GLU) is also added in energy drinks such as CRB [9] and high content of GLU has been demonstrated to produce an oxidative stress in different trials and it is also related to several diseases [10,11,12]. Therefore, it is needed to evaluate the glucose role in the biological activity of energy drinks, comparing CRB to SFRB.

Nutrition is closely related to science since population intakes food to prevent certain diseases. A nutraceutical is any substance that is a food or a part of a food and provides medical or health benefits, including the prevention and treatment of disease. A nutraceutical substance should not be toxic and it should be able to prevent toxicity, avoid genetic oxidative damage, modulate the epigenome marks and induce cell death in a programmed manner in tumour cells, and maintain the epigenome marks in normal cells [13].

Toxicity, antitoxicity, genotoxicity, antigenotoxicity and life and healthspan in vivo assays were carried out in order to characterise the safety and nutraceutical potential of CRB, SFRB, TAU and GLU, establishing their effects on larvae viability, somatic mutations and how they affect life expectancy in *Drosophila melanogaster* model, respectively. *Drosophila* model organism has been used as a reliable model to evaluate the toxicity, genotoxicity and other degenerative process [14,15] and the Somatic Mutation And Recombination Test (SMART), based on the genetic alterations produced in the cells of imaginal discs of the larvae, has been shown to be able to detect genotoxic activity of various compounds with different chemical structures and complex mixtures [16,17].

Regarding in vitro assays, the human leukaemia cell line HL-60 is widely investigated as a model for inducible cell differentiation. This phenomenon might affect the cell ability to proliferate, and thus their immortality, with the appearance of apoptosis [18]. Compounds capable of inducing differentiation and apoptosis in tumour cell lines are candidates to act as chemopreventive agents against cancer [19,20]. Apoptosis is featured by the degradation of genomic DNA into internucleosomal fragments [21]. In addition, apoptosis may be assessed in single cell gel electrophoresis (SCGE) since this assay is capable of measuring DNA breaks and genotoxicity in single cells [22]. Chemopreventive therapies are also related to DNA methylation status in transposable sequences [23] and it is known that environmental exposures to nutritional and chemical factors could alter the epigenome pattern [24].

The aim of this study was to evaluate the biological effects of CRB, SFRB, TAU and GLU in order to check the food safety and the nutraceutical potential of these compounds, characterising different endpoints: (i) Assays of toxicology, antitoxicology, genotoxicology and life expectancy were performed in individuals chronically treated with the tested compounds in the *Drosophila melanogaster* model organism; (ii) The in vitro chemopreventive activity of testing compounds was determined by assessing their cytotoxicity, the proapoptotic DNA-damage capability to induce internucleosomal fragmentation, the strand breaks activity and the modulator role on the methylation status of genomic repetitive sequences of HL-60 promyelocytic cells.

## 2. Results

### 2.1. Toxicity/Antitoxicity

Toxicity and antitoxicity results are shown in Table 1. TAU exerted toxic effects in *Drosophila* larvae at all assayed concentrations, except for the lowest one and GLU provided this toxic effect when larvae were treated with 87.5 and 350 mM GLU. CRB showed a similar pattern to GLU being toxic the two-highest concentrations. Contrarily, SFRB were toxic at all assayed concentrations, except for the two-highest concentrations. Although all tested compounds were toxic in some extent, the lethal dose 50 (LD_50_) standardized parameter was not reached at any of the significant toxic concentration.

Regarding antitoxicity assay, all substances showed some degree of capability to protect larvae population against the oxidative stress of genotoxicant hydrogen peroxide. SFRB, TAU and GLU provided antioxidant effects in a Gaussian-like manner, being antitoxic all the tested GLU concentrations, except for the lowest one. As regards CRB, this beverage exerted antitoxic activities at the three-lowest concentrations in a dose negative response manner.

### 2.2. Genotoxicity/Antigenotoxicity

**Figure 1:**
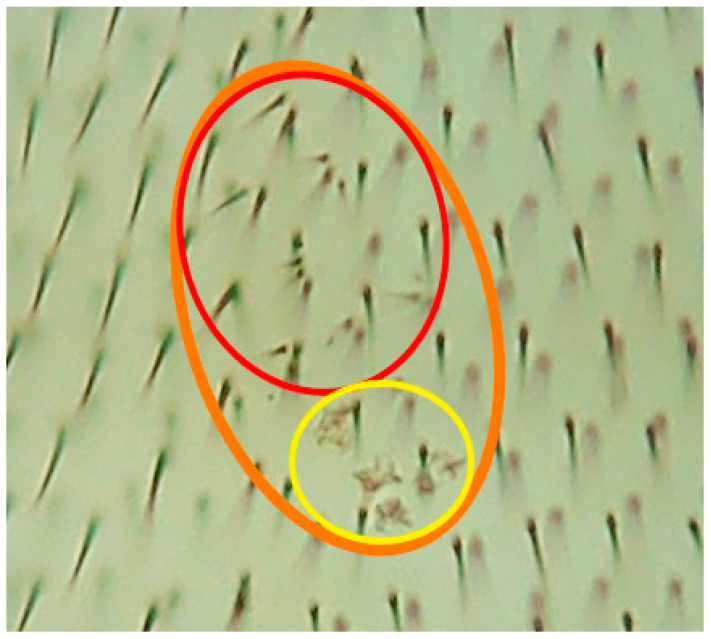
Representative homemade photograph was taken showing the different kind of hairs scored in the tested compounds: twin spot (**orange circle**); *mwh* mutation (**red circle**); *flr3* mutation (**yellow circle**); left being wild type single trichome hairs.

Genotoxicity and antigenotoxicity results performing the SMART assay are shown in Table 2 and representative photograph of different types of hairs are depicted in Figure 1. The positive result obtained in the positive control after applying the binomial Kastenbaum-Bowman test, validated the accuracy of the SMART.

A Kastenbaum-Bowman test revealed negative results for both tested concentrations of SFRB, 1 mM TAU and 350 mM GLU. This test provided inconclusive results in both concentrations of CRB, 32 mM TAU and 11 mM GLU. The inconclusive results were resolved applying the Mann-Whitney test obtaining negative statistically values. Therefore, none of the tested compounds were genotoxic at any concentrations assayed. On the other hand, the IP values were calculated to determine the antigenotoxic potential of each compound when the clones per wings of the total spots from antigenotoxicity assay were different from the positive control (0.425) after applying the Kastenbaum-Bowman test (positive results). All tested compounds resulted in protecting *Drosophila* larvae against hydrogen peroxide in a negative dose-dependent manner, except for 4 mg/mL CRB which provided 0.375 clones per wing being statistically similar to the positive control. The IP values obtained were as follows: 69.4% for 130 mg/mL CRB; 62.8% and 29.4% for 0.625 and 20 mg/mL SFRB, respectively; 70.6% and 38.1% for 1 and 32 mM TAU, respectively and 76.5% and 52.9% for 11 and 350 mM GLU, respectively.

### 2.3. Lifespan

**Figure 2:**
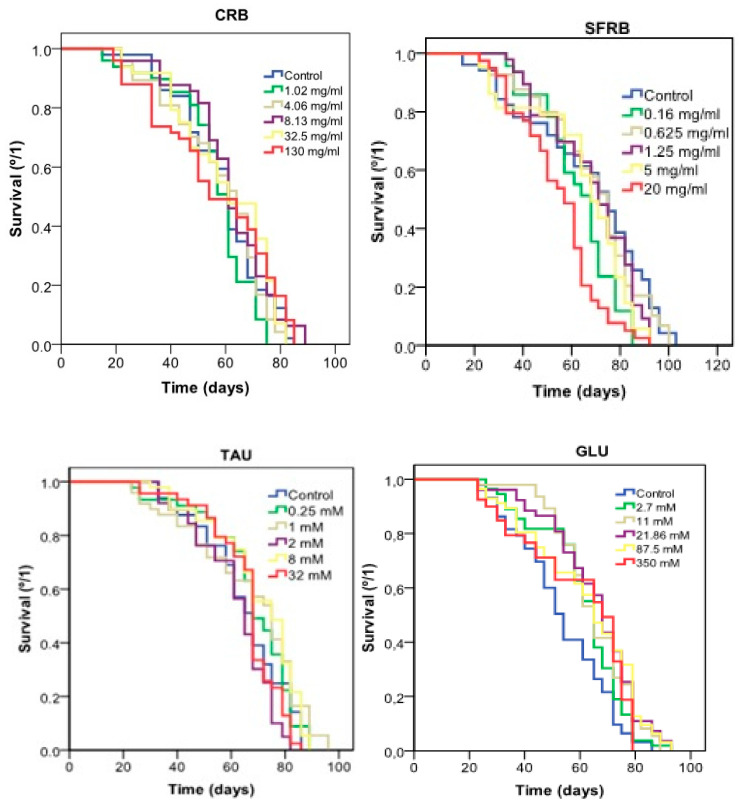
Effect of CRB, SFRB, TAU and GLU supplementation on the lifespan of *Drosophila melanogaster*.

Figure 2 and Table 3 show the lifespan results obtained in *Drosophila melanogaster* experimental model treated with CRB, SFRB, TAU and GLU, resulting in an significant decreasing (roughly 18%) of the life expectancy in flies treated with 20 mg/mL of SFRB. However, CRB, GLU and TAU did not significantly differ from their concurrent control.

Regarding healthspan showed in Table 3, 4.06 and 130 mg/mL CRB were able to decrease the quality of life around 27–29% with respect to the control. In addition, 5 mg/mL SFRB also reduced 20% the healthspan in *Drosophila*. By contrast, 350 mM GLU was able to increase the quality of life in 34%.

### 2.4. Cytotoxicity

**Figure 3:**
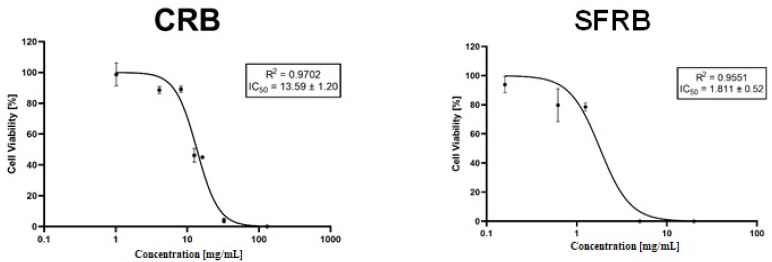

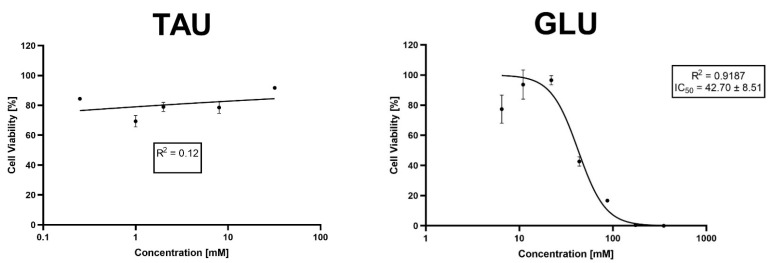
Cytotoxic effects of CRB, SFRB, TAU and GLU. Viability curves at 72 h of treatment. Data are expressed as mean ± SD, including IC_50_ value.

Our cytotoxicity assays reported that all tested substances were chemopreventive compounds reaching IC_50_ at lowest tested concentrations, except for the TAU where IC_50_ was not found as it is depicted in Figure 3.

### 2.5. Internucleosomal DNA Fragmentation

**Figure 4:**
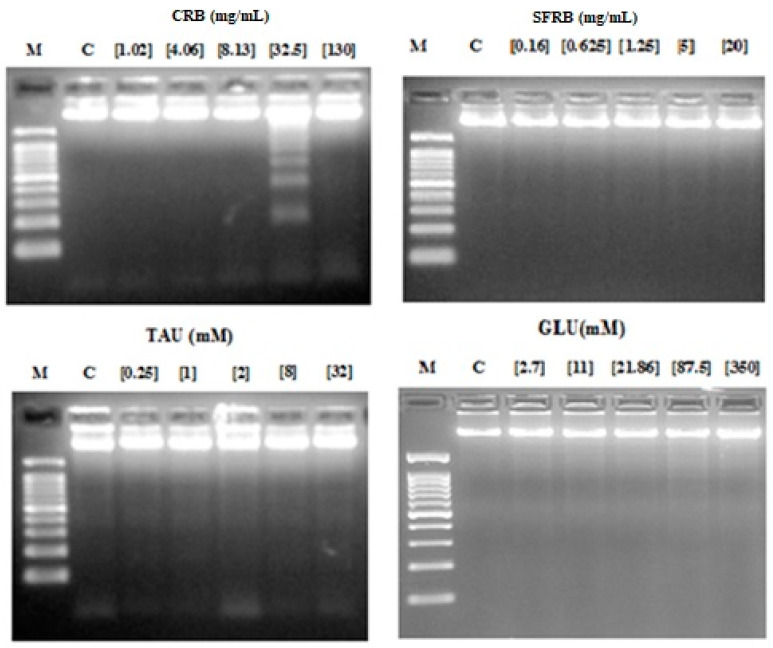
Internucleosomal DNA fragmentation after 5 h of treatment with CRB, SFRB, TAU and GLU. Letters M and C mean weight size marker and negative control respectively.

According to internucleosomal DNA fragmentation assay, 32.5 mg/mL CRB was able to induce the typical ladder pattern showed in apoptotic cells. Nevertheless, SFRB, TAU and GLU did not induce DNA fragmentation in HL-60 cell line (Figure 4).

### 2.6. Comet Assay

**Figure 5:**
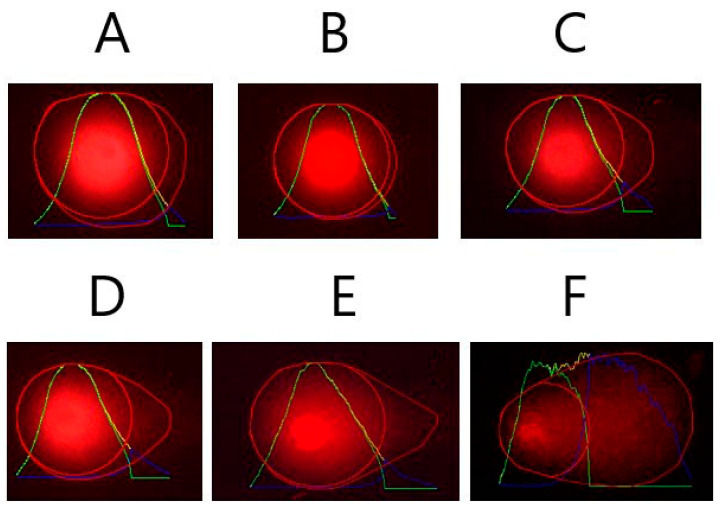
Representative photographs obtained in our SCGE assay for each treatment once Tail Moment (TM) parameter was analysed using OpenComet plugin for ImageJ Software. (**A**) negative control (TM = 0.81), (**B**) treated HL-60 cells with SFRB (TM = 0.125), (**C**) treated HL-60 cells with TAU (TM = 2.49), (**D**) treated HL-60 cells with RB (TM = 3.57), (**E**) treated HL-60 cells with GLU (TM = 5.51) and (**F**) positive control showing the hedgehog pattern (TM = 82.74).

**Figure 6:**
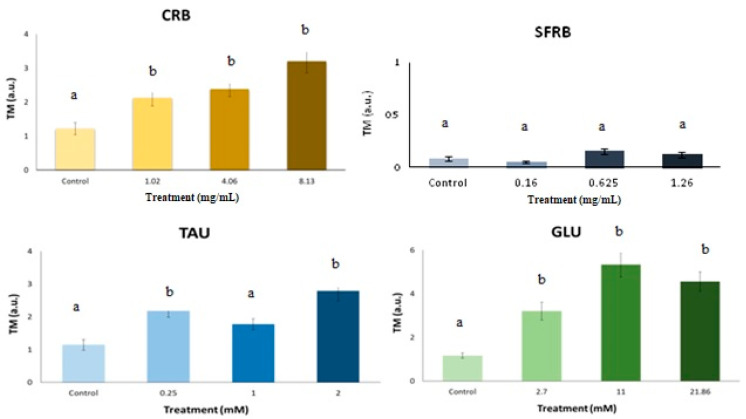
Alkaline comet assay (pH < 13) of HL-60 cells after 5 h-treatment with different concentrations of CRB, SFRB, TAU and GLU. DNA migration is reported as mean TM. The plot shows mean TM values and standard errors. Different letters mean different values after one-way ANOVA and post hoc Tukey’s test.

Figure 5 shows representative photographs of Tail Moment (TM) obtained in (A) negative control (TM = 0), (B) treated HL-60 cells with GLU (TM = 5) and (C) positive control (with a hedgehog pattern). Our SCGE assay revealed that CRB and GLU significantly induce TM values higher than the concurrent control producing DNA damage in HL-60 cell line as well as 0.25 and 2 mM TAU. However, SFRB was not able to induce DNA strand breaks as it is shown in Figure 6. The concentrations used in this SCGE assay were determined according to the results obtained in the previous cytotoxicity assay.

### 2.7. Methylation Status

**Figure 7:**
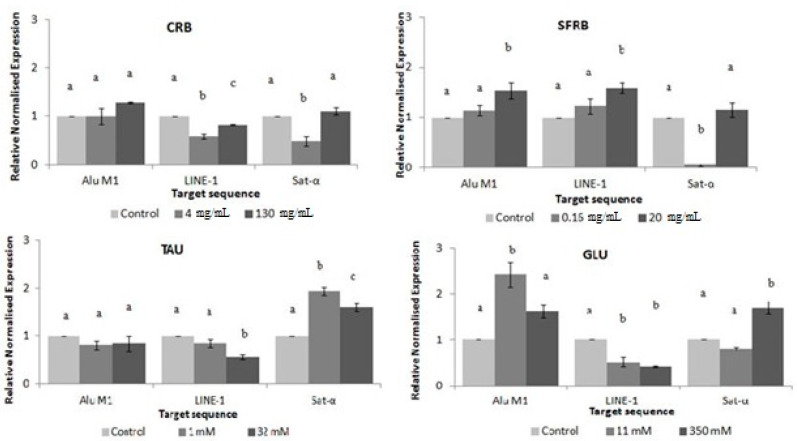
Relative normalised expression data of each repetitive element. Different letters are associated with different means applying One-Way ANOVA test and post hoc Tuckey’s test.

Figure 7 shows the relative normalised methylation status (RMS) of the three repetitive sequences (LINE-1, Alu M1, and Sat-α) in HL-60 cell line treated with the tested compounds. CRB and TAU hypomethylated LINE and Sat-α repetitive elements showing a similar pattern in satellite sequences. However, while CRB was able to reduce the methylation status of LINE sequences at the lowest concentration assayed, TAU reduced this methylation status at the highest one. Both tested concentrations of TAU significantly hypermethylated satellite alpha sequences. The highest assayed concentration of SFRB hypermethylated Alu and LINE repetitive elements, whereas the lowest one reduced the methylation status of Sat-α sequences. Finally, 11 mM GLU hypermethylated Alu sequences and hypomethylated LINE elements and whereas 350 mM GLU reduced the methylation status of LINE repetitive element in HL-60 cell line, this concentration hypermethylated satellite alpha sequences.

## 3. Discussion

The arrival of the “genomic era” has raised the concept of the nutrigenomic studies, which aim is to expose the relationship between nutrition and genome to supply a scientific basis for improved public health through dietary habits [26]. aking into account that TAU is one of the main bioactive additives of CRB and SFRB, its evaluation is necessary to understand the physiological effects of both energy drinks.

We performed in vivo assays ascertaining on toxicity/antitoxicity and genotoxicity/antigenotoxicity in the SMART model as well as on lifespan and healthspan using the same *Drosophila* genetic background. We also carried out in vitro assays assessing on the cytotoxicity, internucleosomal DNA fragmentation, DNA damage and methylation status using HL-60 human leukaemia cell line. None information has been found in scientific databases about CRB and SFRB related with our assays. Contrarily, there are some data available about TAU and GLU. Therefore, it is the first time that CRB and SFRB are studied with this scope so our results will provide some important data about the biological effects of these worldwide consumed compounds.

The effect of energy beverages, glucose and taurine on *D. melanogaster* in vivo model *Drosophila* is considered an accurate in vivo model to study human diseases and further substantial contributions in this sense are expected [27]. To our knowledge, this is the first attempt to characterise the genotoxic effect of CRB and SFRB using the *Drosophila* in vivo model and HL-60 in vitro model. Experimental doses of TAU mimicking the concentration used in CRB were tested as well as GLU in this study.

The toxicity results obtained suggest that CRB, TAU and GLU are significantly toxic at most assayed concentrations, showing the beverage and GLU a positive dose-dependent manner. Contrarily, SFRB significantly resulted in being toxic at the three lowest concentrations in a negative dose-dependent manner. CRB (10 mg/kg/day, during 4 weeks) was demonstrated to induce distorted architecture of pancreatic sections of rats and the level of glucose in blood was increased [28] increase the oxidative stress altering the superoxide dismutase in liver, kidney, testes and brain but had not effect in heart resulting toxic the consumption of CRB in rats [29]. Moreover, the toxicity of CRB was reported in rabbit treated during 21 days at doses lower than the manufacturer’s recommended equivalent for human intake [30]. Our CRB results are consistent with the toxicity mentioned above up to a point since de classical toxicity parameter LD_50_ has been not reached at any tested concentration. Bigard [31] demonstrated that although TAU was toxic in encephalopathy diseases, its toxicity was low besides, energy drinks were related to adverse effects due to their components. Besides, a great deal of incidents related to energy drinks toxicity is underestimated by the National Poison Data System [32]. Our results agree with those obtained since TAU could provide the toxicity of CRB as well as GLU which was demonstrated to be toxic in several in vivo trials triggering oxidative stress [33] being in agreement with our results.

Nevertheless, the standardized lethal dose 50 (LD_50_) was not reached by any concentration and substance assayed in this study thus they are in agreement with TAU security demonstrated by Shao and Hathcock [34] both in human and animals trials using lots of concentrations. Moreover, we used a wide range of concentrations taking into account the *Drosophila* consumption and weight in order to be able to extrapolate the results to humans. If we consider a person whose daily intake was about 2 L of CRB, the concurrent concentration would be 4 mg/mL for RB and 1 mM TAU in *Drosophila* organism model [35]. If these concentrations are fixed, CRB would not be toxic at all. Regarding TAU, the toxicity could be due to the organism model used since Massie et al. [36] demonstrated that TAU could be act as a larvicide in a mechanism which only would occur in insect, although LD_50_ was not found as it was mentioned before. Therefore, the toxicity observed in our study could be explained in this sense. We hypothesise that the lack of toxicity of 4 mg/mL CRB could be due to others additive such as GLU or caffeine [37], among others.

Ceriello et al. [10] revealed that GLU produces an oxidative stress which is in agreement with the certain toxicity found in our GLU results. In addition, in this study was demonstrated that this oxidative stress in human endothelial cells triggers the induction of antioxidant enzymes which could be the responsible for the antioxidant activity of GLU found in *Drosophila* at all concentrations except for the lowest one. Our results provided evidences of the antioxidant activity of TAU at lowest concentrations. This result is in agreement with the hypothesis that TAU is able to block the ROS generation related to the toxicity produced by oxidative stress [38]. TAU was able to reduce the stress oxidative and apoptosis led by temperature in pufferfish, enhancing the activity of antioxidant enzymes [39]. Similar results to TAU antioxidant and antiapoptotic effects were found by Abdel-Daim et al. [40] in rats. However, TAU was demonstrated to be a low antioxidant compound in in vivo (rats) assays some years ago [41]. Therefore, the antioxidant effects of CRB in *Drosophila* may be due to the TAU and GLU biological synergic activity.

Research using *Drosophila* has provided seminal insights into gene function which are relevant to human health [42]. The genomic stability (lack of genotoxicity) observed in *Drosophila* with all the compounds assayed confirmed their safety. The inconclusive results obtained in some tested concentrations mean that the null hypothesis is accepted thus it is assumed that there is no difference in the mutation frequency between control and treated series. However, the alternative hypothesis is also accepted what means that the mutation frequency is not significantly 2-fold lower than the postulated increased frequency. No significant differences were found between the tested concentrations and the negative control when inconclusive results were resolved applying the Mann-Whitney U-test [43].

Direct studies on genotoxicity of TAU, CRB and SFRB are barely available because the epidemiological data always shows protective anticancer health properties such as antioxidant or antigenotoxic activity. These results agree with the previously reported by Cozzi et al. [44] who determined that 1 mM TAU was not able to induce neither chromosome aberrations nor sister chromatid exchanges in CHO cells. Lymphocytes cells treated with 20–50 mM TAU showed the similar number of sister chromatid exchange values than the concurrent control [45]. TAU and/or caffeine could account for the absence of genotoxicity of CRB [37]. GLU is involved in DNA damage processes lead by glycated products provoking base modification, strand breaks and apurinic/apyrimidinic sites in DNA [46]. Sucrose and GLU were determined as genotoxic compounds in the colon epithelium of rats at higher concentration (30% GLU) than the human intake using the mutation frequencies of the *E. coli* lambda and the level of bulky DNA adducts tests [47]. On basis to literature, the lack of genotoxicity found in our GLU results may be due to the fact that the mutagenicity showed by GLU is related to Maillard reaction products when the sugar is heated in presence of other compound.

It was demonstrated the antigenotoxic effect of TAU on aluminum sulphate-induced DNA damage in human peripheral lymphocytes protecting the DNA against the stress oxidative induced by this coagulant [48]. In addition, 5–20 mM TAU was reported as DNA-protected concentrations against oxidative damage in calf thymus DNA [49] and reduced genotoxicity activity of some drugs (benznidazole, cyclophosphamide and metronidazole, among others) in mice [50]. Our glucose results suggested that this sugar is able to protect the DNA of *Drosophila* against oxidative damage caused by hydrogen peroxide in large extent. All information available in scientific database on glucose DNA protection is related to cause this damage instead of protecting it and the information found about CRB and SFRB is scarce in connection with our assays.

*D. melanogaster* is an excellent model for the study of aging because adults show many similarities with the cellular senescence observed in mammals [51]. This is the reason why this particular model is frequently used to understand the relationship between nutrient metabolism and aging mechanisms [52]. As far as we know, the anti-ageing and anti-degenerative effects of CRB were assayed for the first time using *D. melanogaster* in our study. We demonstrated that none of the tested substances was able to increase the life expectancy of fruit flies. However, CRB decreased the quality of life of this model organism. TAU had not any influence on quality of life of *Drosophila* although an increase of hemoglobin levels was associated with high amount of TAU in human patients thus the quality of life could be improved [53]. Furthermore, it is used as antioxidant in order to improve the quality of life reducing the adverse effects of some disease such as diabetes mellitus [54]. The absence of lifespan improvement was reported long time ago as TAU (0.05 to 0.20 M) had no influence on adult lifespan of *Drosophila* [36]. In fact, 1.6% TAU was demonstrated to decrease the lifespan of *Drosophila*. Conversely, subsequent reports on *Drosophila* lifespan demonstrated that TAU increased the estimated mean values of survival at 8–24 mM [53]. Furthermore, Smith et al. [55] reported that TAU could also increase the lifespan of *Drosophila* [56]. TAU reduced the negative effect of tunicamycin on *C. elegans* lifespan restoring its normal values [57]. A multi-country epidemiological meta-analysis revealed that dietary TAU intake is negatively correlated with mortality from ischemic heart disease [57,58]. Nevertheless, Ito et al. [59] demonstrated that tissue TAU depletion shortens lifespan concomitant with acceleration in tissue aging. Despite a lifespan extension was not induced by GLU in our assays, it is well-known that GLU restriction can extend the lifespan of normal cells [52] including *Drosophila* [60] and the information found in scientific databases is restricted in this sense. Although CRB did not increase the lifespan, its safety has been demonstrated again. Conversely, SFRB decreased the lifespan of flies at the highest tested concentration in spite of the caloric restriction which flies were undergone to.

The in vitro evaluation of the anti-cancer properties of nutraceutical compounds or foods is the first step of a large pathway to obtain suitable conclusions to be extrapolated to human [37]. We determined the potential chemopreventive and genotoxic effect of CRB, SFRB, TAU and GLU on a human cancer cell model (HL-60 cell line).

CRB, SFRB and GLU showed cytotoxic effects following a positive dose-dependent response, being the inhibitory concentration 50 (IC_50_) 13.59 mg/mL, 1.81 mg/mL and 42.70 mM, respectively. On the other hand, TAU did not show cytotoxic activity in HL-60 cells at any assayed concentration, and only the 2 mM concentration induced a decrease of cell proliferation. Jeon et al. [61] reported that TAU increased cell proliferation although they were not cancerous and Heidari et al. [62] demonstrated the protective effects of TAU against isoniazid and its intermediary metabolite hydrazine cytotoxicity in rat hepatocytes. These reports support our findings, suggesting that TAU is not cytotoxic since it may even protect HL-60 cells against toxic damage. Among the other tested compounds, they showed a similar pattern inhibiting around 100% of the cell growth at the second-highest concentration, which does not occur with none of the tested concentrations of TAU. In addition, our result fit in with those obtained by Chen et al. [63] who demonstrated that TAU up-regulate the taurine-upregulated gene 1 (TUG1) which serves an oncogenic role in the development of several tumors. Therefore, TAU would not be the responsible for the cytotoxic activity showed by CRB, being GLU one of the bioactive compounds involved in that effect. It has been previously described that GLU deprivation induces cell death in human breast carcinoma cells [64] and a high-rate glycolysis is related to promote cancer cell survival [65]. Therefore, our GLU result is not in agreement with these assumptions being a noteworthy finding. Nevertheless, 30 mM GLU cytotoxicity was demonstrated in human umbilical endothelial cells inducing stress oxidative by the generation of free radicals [66] being in concordance with the idea based on the hyperglycemia cytotoxicity in a positive dose-dependent response [67] in normal cells. On the other hand, fructose has been considered as a chemopreventive additive [68] while there is controversial information according to caffeine cytotoxic properties. We hypothesise that CRB sugar content could provide the chemopreventive potential of this energy drink and the artificial sugar sweetened could act similarly in SFRB.

The degradation of genomic DNA into internucleosomal fragments was proposed as a major mechanism affecting cancer cell apoptosis. Figure 4 indicates that DNA fragmentation was observed in the sec highest concentration of CRB (32.5 mg/mL) whereas none of the tested concentrations of SFRB, TAU and GLU showed this ladder pattern. Thus, these three compounds are not able to induce apoptosis in HL-60 cell line and these results fit in with the studies of Chen et al. [69] and Takatani et al. [70] who proved that TAU has no effect on induction of cell apoptosis. Chang et al. [71] demonstrated that DNA protecting compounds were able to prevent apoptosis which is in agreement with our results. ROS are essential mediators of apoptosis which eliminates cancerous and other life-threatening cells. Excessive DNA protecting substances could interfere with this mechanism [72]. Our findings suggest that the cytotoxicity observed in CRB may be conducted by proapoptotic mechanisms in some extent. This type of death cell is not induced in HL-60 cell line treated with TAU because of the lack of cytotoxicity showed by this additive. According to GLU, it has been demonstrated to induce DNA fragmentation in normal cells such as proximal tubular cells treated with 10% GLU under hyperglycemia conditions [73] and in cultured human umbilical vein endothelial cells fed with 30 mM GLU [74]. However, our results fit in with those obtained by Cao et al. [65] and Vaughn and Deshmukh [75] who reported that hyperglycemia environment stabilised tumour cells and block apoptosis mechanisms. We suggest the fructose as one of the main responsible for this proapoptotic mechanism induced by CRB since fructose was able to induce DNA fragmentation [76] once any tested concentrations of caffeine did not reach IC_50_ in a similar assay [37].

We performed alkaline SCGE in order to detect DNA damage [77], which are widely used to determine whether cells are triggering apoptotic and/or necrotic pathways [78]. It is assumed that apoptosis occurs when treatments induces a TM > 30 (hedgehog pattern) whereas control cells remain lower than 2 (no tails). On the contrary, necrosis shows a short comet-tail pattern since the majority of the damaged DNA remains in the comet head [79]. Our results showed that the damage induced by CRB and TAU in HL-60 cells was characterised by necrosis (short tails, TM < 3.2, Figure 6). These results agree with our cytotoxicity and DNA fragmentation assays, demonstrating that CRB, TAU and GLU induced cell death in HL-60, probably mediated by a necrotic pathway, when appropriate. The apoptotic way observed in HL-60 cell line treated with CRB also are in agreement with these comet results since the concentration which induced apoptosis was not assayed on comet assay due to the fact that it was highly cytotoxic and none cell was found. Our results fit in with Mochizuki et al. [80] who reported that tumour necrosis factor alpha (TNF-α) increases the amount of the TAU transporter and its affinity, resulting in an increase of the intracellular TAU level. These tested compounds exhibited the same DNA damage pattern: class 1; TM between 1 and 5 according to Fabiani et al. [81]. The lack of in vitro genotoxicity induced by TAU was also observed previously by Ahmad et al. [82] where the tail length of cell treated with TAU was similar to the concurrent control in rats. In normal cells such as human endothelial cells, 30 mM GLU was demonstrated to induce single strand breaks [83] and necrosis mechanisms in peripheral blood monocytes [84].

The genome instability is triggering by a globally hypermethylated status and the repetitive elements are highly methylated in somatic normal cells. However, cancer cells are generally hypomethylated, especially transposable elements, contributing to genome instability [85,86]. LINE-1(Long interspersed nuclear elements), Alu M4 and Sat-α repetitive elements are studied in the present work thus 32% of the genome has been analysed searching for methylation levels on transposable elements [87]. LINE-1 is non-random distributed by accumulating in G-positive bands (AT-rich regions) whilst Alu elements are included in non-coding GC-rich regions. On the other hand, Sat-α repetitive elements are AT-rich regions present in centromere [88,89].

Our results of DNA methylation status showed a different response with respect to the repetitive sequences screened. The methylation status of Alu sequences was significantly increased at 20 mg/mL SFRB and 11 mM GLU. CRB and GLU generally hypomethylated LINE-1 as well as 4 mg/mL of CRB and 0.16 mg/mL of SFRB hypomethylated Sat-α repetitive element, whereas SFRB hypermethylated LINE sequences at the highest concentration. In addition, TAU induced hypomethylation in LINE-1 at 32 mM. Contrarily, both tested concentrations of TAU hypermethylated Sat-α in a significant negative dose-dependent manner and this results fit in with Lleu and Huxtable [90] who proved that TAU increases the degree of phospholipid methylation in vivo in cerebral cortical synaptosomes of developing rats. This hypermethylation could be considered as a benefit since Sat-α represents the main DNA component of every human centromere [88,91]. Although the information related to the modification of the methylation status of repetitive elements induced by GLU is rather scarce on scientific database, its ability to modify the methylation pattern has been proved since hyperglycemia is associated with epigenetic changes in the promoter of the nuclear factor kB subunit p65 in aortic endothelial cells in mice and induces proinflamatory cytokines modulating the histones [92,93]. Glucose restriction is also related to the induction of DNA methylation changes in normal and immortalised cells acting as a therapy in cancer cells [94].

It has been demonstrated that the expression of satellite sequences is associated with a hypomethylation triggering cancer cells. Therefore, methylation process in satellite sequences is a potential mechanism for silencing its satellite expression in transformed cells [95]. On the other hand, human therapies against cancer are based on hypomethylation agents since this therapy is highly related to gene silencing thus this fact could activate tumour suppressor genes and be a positive highlight [96]. These are the reasons why CRB, TAU and GLU could be proposed as hypomethylated agents since they induce a global hypomethylation in some extent and hypermethylated satellite sequences. Moreover, TAU and GLU could be suggested as the responsible compound for the CRB properties. The hypermethylation showed by SFRB could be considered as a benefit since LINE-1 has been related to C-met oncogene what would be silenced [97].

## 4. Materials and methods

### 4.1. Samples

The energy drinks selected for this study, CRB and SFRB, was bought in a local market, lyophilised (SCAI, University of Córdoba, Córdoba, Spain) and stored at room temperature in a dark and dry atmosphere until being used. Furthermore, two single bioactive compound of these beverages were analysed, TAU (2-aminoethanesulfonic acid, Cat. No. 107-35-7, Sigma-Aldrich, St. Louis, MO, USA) and GLU (Cat. No. 50-99-7, Applichem Panreac, Barcelona, Spain). In order to make comparable with a 330 mL daily intake of energy drinks consumption in human (70 kg human body weight), the concentration ranges of compounds used in the different assays were calculated regarding the average daily food intake of *D. melanogaster* (1 mg/day) and the average body weight of *D. melanogaster* individuals (1 mg) [98].

### 4.2. Fly Stocks

Two *Drosophila melanogaster* strains with genetic markers that affect the wing-hair phenotype were used: (i) *mwh/mwh*, carrying the recessive mutation *mwh* (multiple wing hairs) [99], and (ii) *flr^3^/In (3LR) TM3, rip^p^se^p^bx^34e^e^s^Bd^S^,* where the *flr^3^* (flare) [100] marker is a homozygous recessive lethal mutation which is viable in homozygous somatic cells once larvae start developing and producing deformed trichomas.

Two strains of *D. melanogaster*, *mwh* (multiple wing hairs) that and *flr^3^* (flare) have been used. Both strains carry hair markers genes on the third chromosomes, being different in the shape and the number of hairs per cell as follows: (i) mwh/mwh, carrying the recessive mutation mwh that produces multiple trichomas per cell instead of one per cell in homozygosis [99]; (ii) *flr3/In (3LR) TM3, rip^p^sepbx^34e^e^s^Bd^S^* is a lethal recessive marker in homozygosis that produces deformed trichomas but is viable in homozygous somatic cells once larvae start the development [100].

### 4.3. Cell Culture Conditions

Promyelocytic human leukaemia (HL-60) immortal cells originated from a female suffering myeloid leukaemia [101] were grown as suspension cultures in RPMI-1640 medium (R5886, Sigma-Aldrich, St. Louis, MO, USA) supplemented with 10% heat-inactivated foetal bovine serum (S01805, Linus, Madrid, Spain), L-glutamine 2 mM (G7513, Sigma-Aldrich, St. Louis, MO, USA) and 1× antibiotic-antimycotic solution from a 100× stock solution containing 10,000 units of penicillin, 10 mg of streptomycin and 25 µg of amphotericin B per mL (A5955, Sigma-Aldrich, St. Louis, MO, USA). Cells were incubated at 37 °C in a 80% humidified atmosphere 5% CO_2_. Cultures were plated at 2.5 × 10^4^ cells/mL density in 10 mL culture bottles and passed every 2 days.

### 4.4. In Vivo Assays

#### 4.4.1. Toxicity and Antitoxicity Assays

Toxicity was assayed according to our standard protocols. CRB and SFRB were tested at five concentrations: 1, 4, 8.12, 32.5 and 130 mg/mL and 0.16, 0.625, 1.25, 5 and 20 mg/mL, respectively. TAU and GLU were tested at 0.25, 1, 2, 8 and 32 mM and 2.7, 11, 21.86, 87.5 and 350 mM, respectively. Negative (H_2_O) and positive (0.15 M H_2_O_2_) toxicant concurrent controls were also assayed. Tested groups consisted of larvae fed with *Drosophila* Instant Medium (Formula 4–24, Carolina Biological Supply, Burlington, NC, USA) supplemented with the assessed compounds concentrations. Emerging adults of all groups were counted and toxicity was determined as the percentage of hatched individuals in each treatment compared with the negative control. Antitoxicity was assessed using the same procedure and experimental concentrations as in toxicity assays, but in combined treatments with 0.15 M H_2_O_2_ and comparing the percentage of emerging adults with the positive toxicant control [102].

#### 4.4.2. Genotoxicity and Antigenotoxicity Assays

The wing spot test was performed to evaluate genotoxicity assays following the standard procedure [14]. Briefly, trans-heterozygous larvae for *mwh* and *flr^3^* genes were obtained by crossing four day-old virgin *flr^3^* females with *mwh* males in a 2:1 ratio. Four days after fertilization, females were allowed to lay eggs in fresh yeast medium (25 g yeast and 4 mL sterile distilled water) for 8 h in order to obtain synchronized larvae. After 72 h, larvae were collected, washed with distilled water, and clustered in groups of 100 individuals. Each group was fed with a mixture containing 0.85 g *Drosophila* Instant Medium (Formula 4–24, Carolina Biological Supply) and 4 mL water supplemented with the different compounds and concentrations assayed and negative (H_2_O) and positive (0.15 M H_2_O_2_) controls until pupae hatching (10–12 days). Adult flies were collected and stored in 70% ethanol until the wings were removed and mounted on slides using Faure’s solution. Mutant spots were assessed in both dorsal and ventral surfaces of the wings in a bright light microscope at 400× magnification. The frequencies of each type of mutant clone per wing (single, large or twin spot) were compared with the concurrent negative control [43]. All inconclusive and positive results (*p* > 0.05) were analysed with the nonparametric U-test of Mann, Whitney and Wilcoxon (α = β = 0.05).

Anter et al. [13] described the antigenotoxicity method conducted in *D. melanogaster*. The same compounds and concentrations were assayed in combined treatment with hydrogen peroxide (0.15 M) acting as concurrent genotoxicant. Single and twin spots per wing were also recorded and compared with the concurrent negative control as described before. Finally, the inhibition percentages (IP) for the combined treatments were calculated as described by Abraham [103]: IP = [(genotoxin alone—combined treatment)/genotoxin alone] × 100.

#### 4.4.3. Chronic Treatments: Lifespan and Healthspan Assays

Flies used in the lifespan assays show the same genetic background those used in the genotoxicity assays in order to compare both result. The treated adults consisted of the F1 progeny from *mwh* and *flr^3^* parental strains produced by a 24 h eggs-lying in yeast medium. The same compounds and concentrations as in the toxicity/genotoxicity experiments were assayed. Lifespan assays were carried out at 25 ºC according to the procedure described by Fernandez-Bedmar et al. [16]. Briefly, synchronized 72 ± 12-h-old trans-heterozygous larvae were washed in distilled water, collected and transferred in groups of 100 individuals into test vials containing 0.85 g *Drosophila* Instant Medium and 4 mL of the different concentrations of the compounds to be assayed. Emerged adults from pupae were collected under CO_2_ anaesthesia and placed in groups of 25 individuals of the same sex into sterile vials containing 0.21 g *Drosophila* Instant Medium and 1 mL of different concentrations of the compounds to be tested. Flies were chronically treated during all their life. The number of survivors was determined twice a week in three different replicates.

### 4.5. In Vitro Assays

#### 4.5.1. Cytotoxicity Assay

Trypan blue exclusion test was used to determine the cell viability exerted by the assayed compounds, according to our standard procedures [13]. HL-60 cells were placed in 96 well plates (2 × 10^4^ cells/mL) and cultured for 72 h supplemented with 5 different concentrations of CRB, SFRB, TAU and GLU which were selected to assess the cytotoxic doses ranging the inhibitory concentration 50 (IC_50_). After culture, cells were stained with a 1:1 volume ratio of Trypan blue dye (T8154, Sigma-Aldrich, St. Louis, MO, USA) and counted in a Neubauer chamber at 100× magnification. The survival percentage of each treatment compared with the control was recorded in three independent replicates. Data are expressed as mean ± standard deviation of the mean (SEM). When appropriate, the IC_50_ values were analysed using simple linear or nonlinear regression fitting curve with the normalised response using GraphPad Software Prism 9 (San Diego, CA, USA).

#### 4.5.2. DNA Fragmentation Status

DNA fragmentation induction was determined as described by Anter et al. (2014) [104]. Briefly, HL-60 cells (1 × 10^6^/mL) were co-cultured with 5 different concentrations of CRB, SFRB, TAU and GLU similar to those used in cytotoxicity assays, during 5 h. After treatment, genomic DNA was extracted using a commercial kit (Blood Genomic DNA Extraction Mini Spin Kit, Canvax Biotech, Córdoba, Spain). Then, DNA was incubated overnight with RNase at 37 °C and quantified in a spectrophotometer (Nanodrop^®^ ND-1000, NanoDrop Technologies, Inc., Wilmington, DE, USA). Finally, 1200 ng DNA were electrophoresed in a 2% agarose gel for 120 min at 50 V, stained with ethidium bromide and visualized under UV light. The apoptosis process is recognised by the appearance of internucleosomal DNA fragments that are multiple of 200 base pairs.

#### 4.5.3. Clastogenicity: SCGE (Comet Assay)

DNA integrity was assayed by SCGE as described by Olive and Banáth [78] with minor modifications. HL-60 cells (5 × 10^5^) in exponential growing phase were incubated in 1.5 mL of culture medium supplemented with CRB, SFRB, TAU and GLU (the three lowest concentrations selected in the cytotoxicity assays) for 5 h in P-12 plates. After treatment, cells were washed twice and adjusted to 6.25 × 10^5^ cells/mL in PBS. Electrophoresis gels were prepared pouring a 1:4 dilution (cells in liquid low-melting-point agarose at 40 °C, A4018, Sigma) into slides. Gels were covered with a coverslip and allowed to solidify at RT for 30 min. Once the slides solidified, the coverslips were carefully removed and slides were bathed in freshly prepared lysing solution (2.5 M NaCl, 100 mM Na-EDTA, 10 mM Tris, 250 mM NaOH, 10% DMSO and 1% Triton X-100; pH = 13) for 1 h at 4 °C. Thereafter, slides were equilibrated in alkaline electrophoresis buffer (300 mM NaOH and 1 mM Na-EDTA, pH = 13) for 20–30 min at 4 °C. Once equilibrated, the slides were underwent electrophoresis (12 V, 400 mA for 8 min) in the dark and were immediately neutralized in cold neutral solution (0.4 M Tris-HCl buffer, pH 7.5) for 10 min. Finally, slides were dried overnight at RT in the dark. Gels were stained with 7 µL propidium iodide and photographed in a DM2500 microscope (Leica Microsystems GmBH, Wetzlar, Germany) at 400× magnification. At least 50 single cells from each treatment were analysed using the Open Comet^TM^ software (OpenComet, GNU General Public License version 3.0, GPLv3) [105]. A one-way ANOVA and post-hoc Tukey’s test with SPSS Statistics for Windows, Version 19.0 (2010, IBM Corporation, Armonk, NY, USA) was applied to determine the effect of the tested compounds on HL-60 cell DNA integrity from the analyses of Tail Moment (TM) data.

#### 4.5.4. Methylation Status of HL-60 Cells

HL-60 cells were treated with different concentrations of CRB, SFRB, TAU and GLU (as selected in SMART assay) for 5 h. Then, DNA was extracted similarly to previously described DNA fragmentation assay. After that, the DNA was converted with bisulphite (EZ DNA Methylation-Gold™ Kit, Zymo Research, Irvine, CA, USA). Bisulphite-modified DNA was used for fluorescence-based real-time quantitative Methylation-Specific PCR (qMSP) using 5 µM of each forward and reverse primer (Isogen Life Science BV, Utrecht, The Netherlands), 2 µL of iTaq™ Universal SYBR^®^ Green Supermix (Bio-Rad Laboratories, Inc., Hercules, CA, USA); it contains antibody-mediated hot-start iTaq DNA polymerase, dNTPs, MgCl2, SYBR^®^ Green I dye, enhancers, stabilizers and a blend of passive reference dyes including ROX and fluorescein) and 25 ng of bisulphite converted genomic DNA.

PCR conditions included initial denaturalisation at 95 °C for 3 min and amplification which consisted of 45 cycles at 95 °C for 10 s, 60 °C for 15 s and 72 °C for 15 s, taking picture at the end of each elongation cycle. After that, melting curve was determined increasing 0.5 °C each 0.05 s from 60 °C to 95 °C and taking pictures.

QMSP was carried out in 48 well plates in MiniOpticon Real-Time PCR System (MJ Mini Personal Thermal Cycler, Bio-Rad) and were analysed by Bio-Rad CFX Manager 3.1 Software. The housekeeping Alu-C4 was used as a reference to correct for total DNA input. Alu-C4 and the target repetitive elements Alu-M1, LINE-1 and Sat-α were obtained from Isogen Life Science (Utrecht, The Netherlands) and their sequences are shown in Table 4. Each sample was analysed in triplicate [106].

The results of each CT were obtained from each qMSP. Data were normalised with the housekeeping Alu C4 using the Nikolaidis et al. [107] and Liloglou et al. [108] comparative CT method (ΔΔCT). One-way ANOVA and post-hoc Tukey’s test are used to evaluate the differences between the tested compounds, repetitive elements and concentrations.

## 5. Conclusions

In summary, this study provides a new corpus data from nutraceutical potential and food safety assays evaluating the toxi/antitoxicity, geno/antigenotoxicity and lifespan in *Drosophila* and the cytotoxicity and clastogenicity, as well as the methylation status of HL-60 cell line. The results shown that CRB, SFRB, TAU and GLU could be considered safe on *Drosophila* taking the LD_50_ parameter into account as well as genotoxicity was not found. On the one hand, according to nutraceutical potential assays related to the protective activity in in vivo assays, both energy drinks, TAU and GLU behaved as antioxidant in some extent against hydrogen peroxide in *Drosophila*. However, promising results were not found in the lifespan assay on *D. melanogaster* even SFRB significantly reduced de life expectancy. On the other hand, chemopreventive potential was shown by all tested compounds, except for TAU in vitro in the HL-60 cell line. CRB induced DNA fragmentation in the second-highest tested concentration although none of the tested compounds displayed significant apoptotic TM values in SCGE test. Therefore, necrotic pathway is the main mechanism from death cell occurs when HL-60 cell line is treated with CRB, GLU and TAU. Finally, TAU and GLU increased the methylation status of satellite sequences in HL-60 cell line providing nutraceutical benefits. On the other hand, CRB globally decreased the methylation pattern which fit in with the fact that some cancer therapies are based on globally hypomethylation. In conclusion, the tested compounds are safe on *Drosophila melanogaster* and CRB could overall possess nutraceutical potential in the in vivo and in vitro model used in this study. Besides, TAU could holistically be one of the bioactive compounds responsible for the biological activity of CRB.

## Figures and Tables

**Table 1:** Toxicity and antitoxicity levels of CRB, SFRB, TAU and GLU in *D. melanogaster*.

CRB (mg/mL)	Survival (%)		SFRB (mg/mL)	Survival (%)		TAU (mM)	Survival (%)		GLU (mM)	Survival (%)	
	Simple Treatment ^(1)^	Combined Treatment ^(2)^		Simple Treatment	Combined Treatment		Simple Treatment	Combined Treatment		Simple Treatment	Combined Treatment
0	100	100	0	100	100	0	100	100	0	100	100
H_2_O_2_	-	38.46	H_2_O_2_	-	58	H_2_O_2_	-	38.46	H_2_O_2_	-	60
1.02	97	49.23 * ^(4)^	0.16	77.65 *	65	0.25	94	40.76	2.7	98	60
4.06	93	47.69 *	0.625	80.3 *	87 *	1	78 *	57.69 *	11	92.65	84.35 *
8.13	88	51.53 *	1.25	74 *	106.68 *	2	77 *	56.9 *	21.86	90.32	88.65 *
32.5	75 * ^(3)^	40	5	93	69 *	8	76 *	40	87.5	70.3 *	79 *
130	59 *	39	20	95.32	53.65	32	78 *	42.3	350	65 *	72 *

^(1)^ Data are expressed as percentage of survival adults with respect to 300 untreated 72-h-old larvae from three independent experiments. ^(2)^ Combined treatments using standard medium and 0.15 M hydrogen peroxide. ^(3)^ Asterisks (*) indicate significant differences (one tail) with respect to the untreated control group and ^(4)^ the hydrogen peroxide control group: * Chi-square value higher than 5.02 [17].

**Table 2:** Genotoxicity and antigenotoxicity of CRB, SFRB, TAU and GLU in the *Drosophila* wing spot test.

Clones Per Wings (Number of Spots) ^(1)^							
Compound	Wings	Small Single Spots	Large Simple Spots	Twin Spots	Total Spots	Mann-Whitney Test ^(2)^	IP (%) ^(3)^
	Number	(1–2 Cells)	(>2 Cells)	m = 5	m = 2		
		m = 2	m = 5				
H_2_O	41	0.147 (6)	0.048 (2)	0	0.195 (8)		
H_2_O_2_ (0.15 M)	40	0.375 (15)	0.05 (2)	0	0.425 (17)+		
SIMPLE TREATMENT							
CRB (mg/mL)							
[4.06]	40	0.2 (8)	0	0	0.2 (8)i	λ	
[130]	40	0.3 (12)	0.025 (1)	0	0.325 (13)i	λ	
SFRB (mg/mL)							
[0.625]	36	0.055 (2)	0.027 (1)	0	0.083 (3)-		
[20]	40	0.125 (5)	0	0	0.125 (5)-		
TAU (mM)							
[1]	37	0.027 (1)	0.08 (3)	0	0.108 (4)-		
[32]	40	0.15 (6)	0.05 (2)	0	0.2 (8)i	λ	
GLU (mM)							
[11]	40	0.125 (5)	0.05 (2)	0	0.175 (7)i	λ	
[350]	40	0.1 (4)	0.025 (1)	0	0.125 (5)-		
COMBINED TREATMENT (*mwh*/*flr*^3^)							
CRB (mg/mL)							
[4.06]	40	0.275 (11)	0.1 (4)	0	0.375 (15) β	λ	
[130]	44	0.11 (5)	0.023 (1)	0	0.13 (6) *		69.4
SFRB (mg/mL)							
[0.625]	38	0.079 (3)	0.053 (2)	0.026 (1)	0.158 (6) *		62.8
[20]	40	0.2 (8)	0.025 (1)	0.075 (3)	0.3 (12) *		29.4
TAU (mM)							
[1]	40	0.125 (5)	0	0	0.125 (5) *		70.6
[32]	38	0.236 (9)	0.026 (1)	0	0.263 (10) *		38.1
GLU (mM)							
[11]	40	0.075 (3)	0.025 (1)	0	0.1 (4) *		76.5
[350]	40	0.175 (7)	0.025 (1)	0	0.2 (8) *		52.9

Statistical diagnosis according to Frei and Wurgler [25]: + (positive), − (negative) and i (inconclusive) vs. negative control; * (positive), Δ (negative) and β (inconclusive) vs. respective positive control; m: multiplication factor. Kastenbaum-Bowman Test without Bonferroni correction, probability levels: α = β = 0.05. ^(1)^ No. of spots in parentheses. ^(2)^ Mann-Whitney test was used when appropriate to resolve inconclusive results. Lambda symbol (λ) means that there are not significant differences with respect to the negative control. ^(3)^ Inhibition percentage values were included when appropriate.

**Table 3:** Effects of CRB, SFRB, TAU and GLU treatments on the *Drosophila melanogaster* mean lifespan and healthspan.

	Mean Lifespan	Mean Lifespan Difference	Healthspan (80th Percentile)	Healthspan Difference
	(days)	(%) ^a^	(days)	(%) ^a^
CRB (mg/mL)				
Control	58.797 ± 2.27	0	37.553 ± 1.96	0
1.02	56.191 ± 2.22	−4.4	34 ± 3.327	−9.3
4.06	57.671 ± 2.548	−2	26.625 ± 2.23 *	−29
8.13	61.59 ± 2.233	4.76	35.4 ± 3.637	−5.6
32.5	59.742 ± 2.637	1.6	32.4 ± 3.04	−13.6
130	56.425 ± 3.06	−4	27.5 ± 2.1 **	−26.6
SFRB (mg/mL)				
Control	67.08 ± 3.6	0	32.4 ± 1.78	0
0.16	62.75 ± 2.5	−6.4	33.75 ± 0.975	4.16
0.625	68.46 ± 3.4	2.2	36.7 ± 3.3	13.27
1.27	68.22 ± 2.7	1.8	36.8 ± 2.21	13.58
5	64.37 ± 3.24	−4	25.75 ± 0.94 **	−20.5
20	54.7 ± 2.7 ***	−18.35	30.25 ± 1.5	−6.6
TAU (mM)				
Control	64.97 ± 2.426	0	37.78 ± 1.94	0
0.25	67.94 ± 2.603	4.57	34.98 ± 3.954	−7.3
1	66.81 ± 3.215	2.81	30.22 ± 1.878	−20
2	61.83 ± 2.25	−4.8	35.357 ± 0.9	−6.4
8	70.142 ± 2.385	7.95	36.8 ± 2.832	−2.6
32	66.04 ± 2.055	1.6	36.57 ± 3.55	−3.2
GLU (mM)				
Control	59.67 ± 2.92	0	32.63 ± 1.49	0
2.7	61.25 ± 2.12	2.65	31.14 ± 1.53	−4.5
11	64.9 ± 2.05	8.76	41 ± 3.4	25.65
21.86	63.21 ± 2.08	5.9	30.66 ± 3.62	−5.8
87.5	60.5 ± 2.5	1.39	29.9 ± 1.96	−8.4
350	59 ± 3.1	−1.2	21.5 ± 0.76 ***	−34

^a^ The difference was calculated by comparing treated flies with the concurrent water control. Positive numbers indicate lifespan increase and negative numbers indicate lifespan decrease. Data are expressed as mean value ±SE. * *p* ≤ 0.05, ** *p* ≤ 0.01, *** *p* ≤ 0.001 significances obtained with the log-rank (Mantel-Cox) test.

**Table 4:** Primers information [85].

Primer	Forward Primer Sequence 5′ to 3′ (N)	Reverse Primer Sequence 5′ to 3′ (N)
ALU-C4	GGTTAGGTATAGTGGTTTATATTTGTAATTTTAGTA (−36)	ATTAACTAAACTAATCTTAAACTCCTAACCTCA (−33)
ALU-M1	ATTATGTTAGTTAGGATGGTTTCGATTTT (−29)	CAATCGACCGAACGCGA (−17)
LINE-1-M1	GGACGTATTTGGAAAATCGGG (−21)	AATCTCGCGATACGCCGTT (−19)
SAT-α-M1	TGATGGAGTATTTTTAAAATATACGTTTTGTAGT (−34)	AATTCTAAAAATATTCCTCTTCAATTACGTAAA (−33)

## Data Availability

The data presented in this study are available on request from the corresponding author.

## Sample Availability

Samples of the compounds are not available from the authors.
